# Research and Forecast Analysis of Financial Stability for Policy Uncertainty

**DOI:** 10.1155/2022/8799247

**Published:** 2022-03-24

**Authors:** Zhiyi Dai, Zhengming Zhou

**Affiliations:** ^1^School of Business, Shanghai University of Finance and Economics, Yangpu District, Shanghai 200433, China; ^2^School of Finance, Shanghai University of Finance and Economics, Yangpu District, Shanghai 200433, China

## Abstract

The instability of financial market will have a great impact on money, bonds, and stocks and affect the economic development of society and people's lives. Therefore, it is very necessary for us to study and predict the financial stability. According to the forecast results, we will analyze and make a series of preparatory measures. First, we make a series of analyses on the structure and significance of policy uncertainty and financial stability. This paper introduces the advantages and disadvantages of the P/L model, the KLS signal method, and the BP neural network model for financial stability early warning, It is clearly pointed out that the BP neural network is more reliable and accurate, Then, the BP neural network, the ant colony algorithm, and the genetic algorithm are used to predict the opening price, closing price, highest price, and lowest price of KDJ index of Cathay Pacific Group's 5-day data. Compared with the real value, we find that the BP neural network is almost the smallest in forecasting the opening price and closing price, or the lowest price and the highest price, and has good stability, which once again proves the feasibility of applying the BP neural network to the research and prediction of financial stability.

## 1. Introduction

The stability of financial market can prevent the possibility of financial crisis. The instability of financial market will bring impact to the securities market, affect people's economic life, and bring bad experience to people's life, so we should always need to care the stability of the market. Uncertainty in economic policy can cause many inconveniences and impacts especially on decision-making including financial intermediaries. By examining the impact on whether the total amount of bank credit will increase. We should have a look on the total amount of credit that will increase or decrease, and then analyze the data owned by the bank entity level. This paper studies and analyzes whether the policy uncertainty will affect the loans of financial intermediaries. The above effects may be attributed to loan demand, which is related to load sheet conditions, or loan supply, which is related to its financial constraints. After analysis and research, we find that it has big bag effect on bank credit become more. Similarly, loan supply factors will obviously have an advantage to determine the impact of goals to a certain extent [1]. Finally, it is concluded that high economic policies will inhibit the growth of overall credit, mainly through bank loans, which will slow down the possible consistency of American economic recovery to a certain extent. This paper investigates the influence of EPU on cross-border mergers and acquisitions. The results show that a high level of domestic EPU will hinder the number and volume of transactions in a certain part, but the host country has moderated this effect positively through its own better conditions. For cross-border M&A, the survey shows that with the same high level of EPU, the target country hinders the entry CBA transactions, while the acquiring country increases the number of its transactions. Finally, an EPU with a larger growth rate will have a lower stock recovery rate compared with the two countries with a smaller growth rate. The results show that if we want to have better transnational investment, we should try our best to reduce the uncertainty of economic policies [2]. Through a macroeconomic model with asset prices, this paper analyzes how several indicators are affected by the central bank's robustness, namely, optimal monetary policy, economic dynamics, and performance. Based on the worst-case model, it is shown that the increase of conservatism preference requires the optimal nominal interest rate to make a series of more positive responses to inflation. The results show that increasing conservatism preference can strengthen the response of current and expected inflation, asset prices, and output to its shocks, but this has almost no impact on commodity demand and financial market shocks [3]. This paper discusses the factors that determine have a question on what can deicide the financial institutions, finds that the policy instability is very important for the proper control of financial system risks, has a significant impact on the target, and it may have an impact on the target through its changed lending behavior and risk-taking ability [4]. This journal points out that economic uncertainty has made a significant contribution to financial risk management, and uncertainty impacts the market and is the decisive factor of banking expansion. Through some dynamic characteristics and forecasting variables, a practical method is constructed, which is mainly based on the price of liquidity factors, to achieve the prediction of interval volatility. It also points out that the uncertainty of China's economic policy and its impact on Taiwan and Hong Kong have a certain impact on their finance [5].We discusses the influence of target on earnings management in Japan and proves that it is negatively correlated with earnings management, that is to say, when EPU is increased, administrators may reduce earnings management. After further analysis of different entities, it is found that the relationship between the two objectives will be less significant under the company with the main bank, which is consistent with the view that the management will have relatively little room to improve the quality of objectives when the EPU is higher. We found similar results when we added analysts to report this indicator. Finally, we find that with the different influencing factors of policy uncertainty, the impact of EPU will also change, and the exposure degree of each enterprise will be different [6]. This paper analyzes and discusses how EPU affects configuration efficiency. The research found that it affected the market value of nearly 25% of the companies, but 90% of them were affected by it and the investment decreased. EPU will distort the signal of capital market, which may aggravate the conflict of interest between managers and investors [7]. We have a question on how the target will influence the investment and capital cost and then try to find an answer. Using a news-based index, looking at 21 countries, we find that a high EPU will reduce the intensity of target negative relationship, and this increase in EPU will reduce the sensitivity of investment to capital cost to a certain extent, whether it is in more opaque countries, enterprises with low analyst coverage or no credit rating. It is said that the answer to the question we started to ask is yes [8].This paper makes an extended research on what kind of influence the target will have on the spread in different ways mechanism of policy by analyzing the relationship between speculators' trust in financial market stability, exchange rate growth and economic development, and the volatility between them. The heterogeneity of error is used to estimate the uncertainty, the results are compared with the literature, and the causes of the financial crisis are obtained. It is found that the low tide of macroeconomic fluctuation will not directly produce the low tide driven by financial stability [9]. This paper points out that the deregulation of financial market will increase the frequency of currency crisis. At present, crisis prediction and financial stability detection are still in their infancy. This paper discusses whether the application of visualization tools based on neural networks will be beneficial to monitoring, mainly self-organizing mapping, which takes time points as benchmarks to intuitively introduce economic indicators and the vulnerability of impending crises. The results show that SOM can be used as a dynamic visualization tool for early warning signals of currency crisis [10].Taking the financial distress data of listed companies in China as research samples, the BP neural network model is trained and tested. The results show that the optimized model improves the prediction accuracy and stability to a certain extent. This study not only have a good outcome on test but also provides new ideas and measures on what we hope it become better [11]. This paper establishes a model related to financial stability. This defines that unregulated private money creation and excessive short-term debt issuance by intermediaries may lead to externalities. Moreover, it can adjust this situation by open market operation. Finally, the model well explains how monetary policy affects practical activities such as bank loans, that is, other trading media can not be controlled by the central bank except banks create money, but this is based on the situation that prices are not adjusted by friction [12]. Faced with the weakness of the existing measurement of financial stability. Too much reliance on macro stress testing, but the “ambiguity” in measurement will not hinder its progress towards the framework. The main features of its framework are strengthening the prudent orientation of supervision; systematically solve the procyclical problem; reduce the burden of measuring financial stability risks in real time; and establish institutional arrangements that draw on the relative expertise of the various authorities involved in safeguarding, in particular financial regulators and central banks [13]. By evaluating the impact of policy uncertainty on China, we find that its increase can reduce investment, mainly for export entry and technological upgrading, and on the contrary, it will reduce real income, which is mainly brought by trade flows and consumers. Through this analysis and research on the export boom before and after China's entry into WTO, it is concluded that the reduction of TPU is a very important policy. We construct a theoretically consistent TPU measure, which not only estimates more than one quarter to about one-third of China's exports to the United States but also estimates a series of effects after removing TP, including welfare benefits for American consumers, and finds that it is similar to the benefits obtained from newly imported varieties [14]. This paper points out that reasons have two. One is the growth of government expenditure, tax revenue, and supervision. Second, the aggravation of political polarization has an impact on its decision-making process and policy choice. Evidence shows that these two reasons have played a certain role in this [15].

## 2. Basic Overview

### 2.1. Policy Uncertainty

Policy uncertainty mainly refers to one of the one of the goals in the article (EPU). In this context, the fluctuation of financial market is a good reflection of the changes in the economic situation at that time, which will have an impact on the expectations of investors and consumers. The composition of EPU in each country is different. The United States mainly includes three parts: news index, tax law failure index, and economic forecast difference index; Europe and China are news indexes. Economic policy uncertainty can be used to explain the change of leverage ratio of listed companies, which has a long impact on it and will aggravate the differentiation of leverage ratio in places with serious financial repression. Time has proved that the rise of policy uncertainty will bring many influences, such as the rise of leverage ratio of state-owned enterprises and the decline of nonstate-owned enterprises. Economic policy uncertainty is closely related to financial market stability, which can increase unemployment rate, increase inflation, and have a negative impact on financial market.

### 2.2. Financial Stability

**Figure 1:**
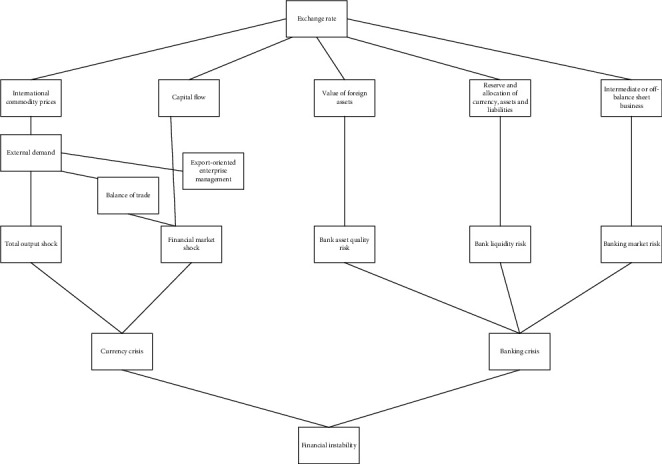
Exchange rate transmission mechanism of financial stability.

Financial stability, most people regard it as the stability of financial institutions, which is mainly the stability of banks, or we can also understand that the main and most financial institutions in the financial system operate steadily without affecting market confidence. On the contrary, financial instability is mainly manifested as the fragility of financial intermediaries or abnormal fluctuations in asset prices, which is also an important basis for monitoring financial stability. Obviously, financial stability is a state, which refers to a state in which a country's financial system runs smoothly without much fluctuation. The exchange rate transmission mechanism of financial stability is shown in Figure 1.

The characteristics of financial stability are

#### 2.2.1. Global

It plays an overall role and is an important provider and defender of some components in the financial institution system. At the same time, banks are the core of financial stability, and its foothold should be a role in maintaining the stability of the whole macro-financial system.

#### 2.2.2. Dynamics

As a state, financial stability is obviously not static and unchangeable, it is constantly changing and developing, and it is a dynamic state. It needs to make corresponding changes with the development of economy and finance. Its internal brother structure adjusts and interacts with each other to form a systematic liquidity system with regulation and control to adapt to the ever-changing financial situation.

#### 2.2.3. Benefit

The stability of the financial system can bring more investment opportunities to enterprises and promote the efficiency of transforming savings into investment.

#### 2.2.4. Comprehensive

Financial stability needs different policies, measures and behaviors, such as monetary policy, financial supervision, and real economy.

**Figure 2:**
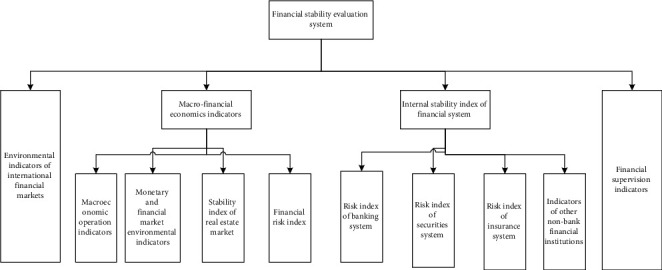
Financial stability evaluation system.

There are two important conditions to judge whether finance is stable: price stability and bank stability. The financial stability evaluation system is shown in Figure 2.

### 2.3. BP Neural Network

BP neural network is one of the most widely used neural network models at present. It is a network model based on error back propagation algorithm. The learning process mainly consists of two processes: forward propagation of signals and back propagation of errors.

In forward propagation, the input of samples is transmitted through the input layer, processed by each hidden layer, and then transmitted to the output layer. If the reality of the output layer does not match the expectation, it is necessary to turn to the back propagation stage of error in time. In the back propagation stage, the input will pass through the hidden layer in a certain form and then reverse transmit to the input layer, and the error will be given to all the units in each layer in the form of equal sharing, so that the error signal means that there will be no gaps in each layer, and this signal can be used as the basis for correcting the weights of each unit.

#### 2.3.1. Transfer Function of BP Neural Network

It mainly adopts a non-linear transformation function-S function, which itself and its derivatives are continuous. The expression of unipolar S-type function is as follows:

(1) $$\begin{array}{c} f\left(x\right)=\frac{1}{1+e^{-x}}. \end{array}$$

The expression of bipolar S-type function is as follows:

(2) $$\begin{array}{c} f\left(x\right)=\frac{1-e^{-x}}{1+e^{-x}}. \end{array}$$

#### 2.3.2. Learning Algorithm of BP Neural Network

This algorithm takes a three-layer perceptron as an example. When the network output is not equal to the expected output, it will output error *E*, which is defined as follows:

(3) $$\begin{array}{c} E=\frac{1}{2}{\left(d-O\right)}^{2}=\frac{1}{2}\sum_{k=1}^{\iota }{\left(d_{k}-o_{k}\right)}^{2}. \end{array}$$

#### 2.3.3. Training Decomposition of BP Neural Network

This process is mainly to adjust two parameters: weight and offset, which are divided into the following two parts:

The output layer calculation formula is as follows:

(4) $$\begin{array}{c} I_{j}=\sum_{i=1}w_{ij}o_{j}+\theta_{j}. \end{array}$$

After random initialization, each weight randomly takes the real number between [–1, 1], and each offset takes the real number between [0, 1], and then starts forward transmission.

(5) $$\begin{array}{c} o_{j}=f\left(I_{j}\right)=\frac{1}{1+e^{I_{j}}}. \end{array}$$

After calculating the output value, we are equivalent to completing the forward transmission part.

For the output layer:

(6) $$\begin{array}{c} E_{j}=O_{j}\left(1-O_{j}\right)\left(T_{j}-O_{j}\right), \end{array}$$

where *E*_*j*_ represents the error value generated by the *J*th node and the output value completed by the *J*th node, it is responsible for completing the task of recording the output value. For example, for the classification of two categories, it is stipulated that 01 represents category 1 and 10 represents category 2. If a record belongs to category 1, its *T*_1_=1, *T*_2_=0.

The hidden layer in the middle is calculated by accumulating the errors of all nodes in the next layer according to the weight, and the calculation formula is as follows:

(7) $$\begin{array}{c} E_{j}=O_{j}\left(1-O_{j}\right)\sum_{k}E_{k}W_{jk}, \end{array}$$

where *W*_*jk*_ represents the weight value from the node *j* of the current layer to the node *k* of the next layer, and *E*_*k*_ represents the error rate of the node *k* of the next layer.

The rules for updating weights are as follows:

(8) $$\begin{array}{c} \Delta W_{ij}=\lambda E_{j}O_{i},\;0\leq \lambda \leq 1,\\ W_{ij}=W_{ij}+\Delta W_{ij}. \end{array}$$

It indicates the speed of learning. The larger its value, the faster the training convergence, but it also has certain disadvantages, that is, it is easy to fall into the local optimal solution. On the contrary, the smaller its value, although the convergence speed is slow, it can approach the global optimal solution step by step.

After completing the above operation update, the bias should also be updated, and its update rules are as follows:

(9) $$\begin{array}{c} \Delta \theta_{j}=\lambda E_{j},\\ \theta_{j}=\theta_{j}+\Delta \theta_{j}. \end{array}$$

When the number of iterations reaches the maximum value, we set or the prediction accuracy of the training set on the network, and the training will be terminated.

#### 2.3.4. Specific Algorithm Flow Chart of BP Neural Network Operation

**Figure 3:**
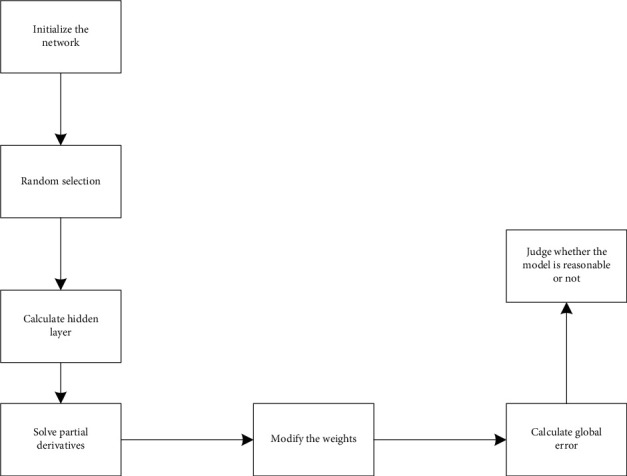
Algorithm flow chart of BP neural network.

The Specific Algorithm Flow Chart of BP Neural Network Operation is as Follows in Figure 3

## 3. Choice of Early Warning Model of Financial Stability

### 3.1. P/L Probabilistic Model

Its expression is as follows:

(10) $$\begin{array}{c} \left\{\begin{array}{c} P\left\{Y=1\right\}=\left(X,\alpha \right),\\ P\left\{Y=0\right\}=\left(X,\alpha \right), \end{array}\right. \end{array}$$

where the probability of 1 means that the financial crisis will occur, 0 means that the financial crisis will not occur, *X* is the inducing factor of the financial crisis, and *α* is a vector of the parameter.

The advantages of this model are easy to understand and simple to operate, and the probability of financial crisis in different countries can be compared from a horizontal perspective. However, the cumulative time and examples show that this model does not consider the differences of different countries repeatedly, which makes its prediction seriously deviated and lacks certain effectiveness.

### 3.2. KLS Signal Method

This signal model is more applied to the early warning of currency crisis, which mainly consists of the following four comprehensive early warning indicators:

(1) Sum all the basic alerts, and the expression is
  (11) $$\begin{array}{c} I_{t}^{1}=\sum_{t=1}^{n}S_{t}^{i},\;I_{t}^{1}\in \left[0,n\right],\;i=1,2,3\ldots n. \end{array}$$
It indicates whether the I index will exceed the safety threshold in *T* period, and its values are only 0 and 1.
(2) Consider the weights of different indicators into early warning indicators
  (12) $$\begin{array}{c} I_{t}^{2}=\sum_{t=1}^{n}\left(SM_{t}^{i}+2SE_{t}^{i}\right),\;I_{t}^{2}\in \left(0,2n\right], \end{array}$$
where *SE*_*t*_^*i*^ is a strong signal and *SM*_*t*_^*i*^ is a weak signal, and the weight value of the strong signal is twice that of the weak signal.
(3) This comprehensive early warning index holds that the outbreak of crisis is not achieved overnight, but a process of accumulation. We regard the time point when the safety threshold is exceeded as the signal of crisis and assume that the time of the model is 8 months, and the expression is as follows:
  (13) $$\begin{array}{c} I_{t}^{3}=\sum_{t=1}^{n}S_{t-s,t}^{i}, \end{array}$$
where *S*_*t*−*s*,*t*_^*i*^ means that he will have an early warning at any time point between [t-s, t], and its values are only 0 and 1, 1 means that it will happen, and 0 means that it will not happen.
(4) Considering the different situations of each early warning indicator, the expression is as follows:
  (14) $$\begin{array}{c} I_{t}^{4}=\sum_{t=1}^{n}\frac{S_{t}^{i}}{w^{i}}, \end{array}$$

where W represents interference ratio, which is mainly for weight, and comprehensive index can be obtained by weighting.

Compared with the P/L model, this model has a better effect and development. However, because many indicators of this model are determined and carried out by historical data, subjectivity will be strong and there will be certain limitations.

### 3.3. BP Neural Network Model

**Figure 4:**
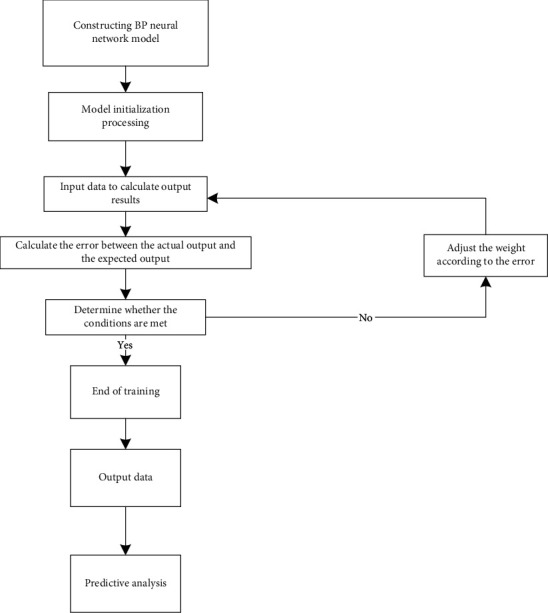
Algorithm flow chart of BP neural network.

The basic process of the BP neural network model mainly includes three parts: initial network creation, learning and training, and early warning. The algorithm flow chart is shown in Figure 4.

#### 3.3.1. Initial Network Creation

This part needs to be combined with the specific problem of determining the specific number of nodes (n, *l*, m) in the input layer, hidden layer, and output layer of the model.

The input layer inputs the data of the hidden layer as *H*_*j*_ , and the value of the hidden layer is *Y*_*k*_ . The calculation formula is as follows:

(15) $$\begin{array}{cc} \left\{\begin{array}{c} H_{j}=x_{1}w_{1j}+x_{2}w_{2j}+\cdots +x_{n}w_{nj}=\overset{n}{\underset{i=1}{\sum }}x_{i}w_{ij};\;h_{j}=f_{1}\left(H_{j}\right),\\ Y_{k}=h_{1}w_{1k}+h_{2}w_{2k}+\cdots +h_{l}w_{lk}=\overset{l}{\underset{j=1}{\sum }}h_{j}w_{jk};\;y_{k}=f_{2}\left(Y_{k}\right), \end{array}\right. & i=1,2,\mathrm{\ldots ,}n;j=1,2,\mathrm{\ldots ,}l;k=1,2,\mathrm{\ldots ,}m, \end{array}$$

where *x*_*i*_ is the input data, *w*_*ij*_ is the connection weight input to the hidden layer, *h*_*j*_ is the passed value, the weight from the hidden layer to the output layer is *w*_*jk*_, *y*_*k*_ represents the output value, and *f*_1_ and *f*_2_ are the conversion functions of two layers outside the input layer. This conversion function generally uses the Sigmoid function, which is a nonlinear function, including log and tan, which have the advantage of converting the values on (−*∞*, +*∞*) to (0, 1) and (–1, 1), respectively.

#### 3.3.2. Carry Out Study and Training

Because the actual output is not completely consistent with the expected output, there are errors between them. Assume their error as *E* and optimize it by using the mean square error minimization method, which is defined as follows:

(16) $$\begin{array}{c} E=\frac{1}{2}\sum_{k=1}^{m}{\left(y_{k}^{*}-y_{k}\right)}^{2}, \end{array}$$

where *y*_*k*_^*∗*^ represents the desired output.

Back propagate the error, and you can get:

(17) $$\begin{array}{c} E=\frac{1}{2}{\sum_{k=1}^{m}\left(y_{k}^{*}-f_{2}\left(Y_{k}\right)\right)}^{2}=\frac{1}{2}{\sum_{k=1}^{m}\left(y_{k}^{*}-f_{2}\left(\sum_{j=1}^{l}h_{j}w_{jk}\right)\right)}^{2}. \end{array}$$

By back propagating to the input layer, you can get:

(18) $$\begin{array}{c} E=\frac{1}{2}{\sum_{k=1}^{m}\left\{y_{k}^{*}-f_{2}\left[\sum_{j=1}^{l}w_{jk}f_{1}\left(H_{j}\right)\right]\right\}}^{2}=\frac{1}{2}{\sum_{k=1}^{m}\left\{y_{k}^{*}-f_{2}\left[\sum_{j=1}^{l}w_{jk}f_{1}\left(\sum_{i=1}^{n}x_{i}w_{ij}\right)\right]\right\}}^{2}. \end{array}$$

In this calculation process, the weight value will constantly change and adjust, so as to achieve a function of reducing error terms. When using gradient descent algorithm to optimize this process, its adjustment amount should be proportional to the negative gradient of error terms, and the following formula can be obtained:

(19) $$\begin{array}{c} \Delta w_{jk}=-\eta \frac{\partial E}{\partial w_{jk}}=-\eta \frac{\partial E}{\partial Y_{k}}\frac{\partial Y_{k}}{\partial w_{jk}},\;j=1,2,\ldots ,l;\;k=1,2,\ldots ,m,\\ \Delta w_{ij}=-\eta \frac{\partial E}{\partial w_{ij}}=-\eta \frac{\partial E}{\partial H_{j}}\frac{\partial H_{j}}{\partial w_{ij}},\;j=1,2,\ldots ,n;\;k=1,2,\ldots ,l, \end{array}$$

where the normal range of the learning rate is (0, 1), and the minus sign in the formula indicates that the gradient shows a downward trend, so that *δ*_*k*_^*y*^=−(∂*E*/∂*Y*_*k*_), *δ*_*j*_^*h*^=−(∂*E*/∂*H*_*j*_), then the expression of the adjustment amount of the weight value can be rewritten into the following form:

(20) $$\begin{array}{c} \Delta w_{jk}=\eta \delta_{k}^{y}h_{j};\\ \Delta w_{ij}=\eta \delta_{j}^{h}x_{i}. \end{array}$$

Therefore, the following results can be calculated:

(21) $$\begin{array}{c} \delta_{k}^{y}=-\frac{\partial E}{\partial Y_{k}}=-\frac{\partial E}{\partial y_{k}}\frac{\partial y_{k}}{\partial Y_{k}}=-\frac{\partial E}{\partial y_{k}}f_{2}^{\prime }\left(Y_{k}\right),\\ \delta_{j}^{h}=-\frac{\partial E}{\partial H_{j}}=-\frac{\partial E}{\partial h_{j}}\frac{\partial h_{j}}{\partial H_{j}}=-\frac{\partial E}{\partial H_{j}}f_{2}^{\prime }\left(Y_{k}\right). \end{array}$$

The output layer can be obtained by the above formula:

(22) $$\begin{array}{c} \frac{\partial E}{\partial y_{k}}=-\left(y_{k}^{*}-y_{k}\right). \end{array}$$

At the same time, the hidden layer formula can be obtained:

(23) $$\begin{array}{c} \frac{\partial E}{\partial h_{j}}=-\sum_{k=1}^{m}\left(y_{m}^{*}-y_{k}\right)f_{2}^{\prime }\left(Y_{k}\right)w_{jk}. \end{array}$$

Substituting formulas (22) and (23) and deriving S function satisfies *f*′(*x*)=*f*(*x*)(1 − *f*(*x*)), the following formula can be obtained:

(24) $$\begin{array}{c} \delta_{k}^{y}=\left(y_{k}^{*}-y_{k}\right)y_{k}\left(1-y_{k}\right),\\ \delta_{j}^{h}=\sum_{k=1}^{m}\left(y_{k}^{*}-y_{k}\right)f_{2}^{\prime }\left(Y_{k}\right)w_{jk}h_{j}\left(1-h_{j}\right). \end{array}$$

Therefore, to sum up, we can get the relevant formula of weight adjustment:

(25) $$\begin{array}{c} \Delta w_{jk}=\eta \delta_{k}^{y}h_{j}=\eta \left(y_{k}^{*}-y_{k}\right)y_{k}\left(1-y_{k}\right)h_{j},\\ \Delta w_{ij}=\eta \delta_{j}^{h}x_{i}=\eta \sum_{k=1}^{m}\delta_{k}^{y}w_{jk}h_{j}\left(1-h_{j}\right)x_{i}. \end{array}$$

Through a series of quantitative analysis, the final weight adjustment formula is as follows:

(26) $$\begin{array}{c} w_{jk}^{t+1}=w_{jk}^{t}+\Delta w_{jk},\\ w_{ij}^{t+1}=w_{ij}^{t}+\Delta w_{ij}. \end{array}$$

Finally, when the actual output meets the expected output, the training can be finished.

#### 3.3.3. (3) Issue an Early Warning

Input data to the trained BP neural network model for prediction, and then according to the output results of the model for a series of analysis of financial crisis and financial stability.

## 4. Feasibility Experimental Study of BP Neural Network

### 4.1. Preprocessing Data Model

For data processing, we generally normalize the index data, so that the later processing is more convenient and the convergence speed can be accelerated. Normalization means that after processing our data in a specified way, it will limit them to the required range. Our input value and target value both stipulate that they can only fall in the range of [0, 1].

The data adopted in this paper come from the securities network software and takes the 5-day KDJ index data of individual stocks Yatai Group. We record the opening price, the highest price, the lowest price, and the closing price data, predict them, respectively, first, and compare the actual output results with the real values to get the results. The forecast data of the opening price of Yatai Group's 5-day KDJ index by different algorithms is shown in Table 1.

Its closing price forecast is shown in Table 2.

The highest price forecast is as follows in Table 3.

Its lowest price forecast is shown in Table 4.

### 4.2. Experimental Comparison Results

**Figure 5:**
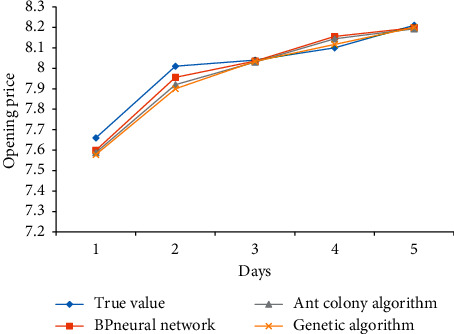
Comparison of opening price forecast results.

**Figure 6:**
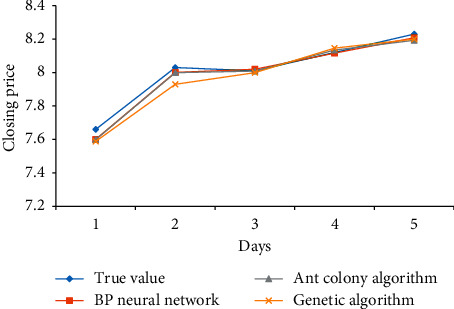
Comparison of closing price forecast results.

**Figure 7:**
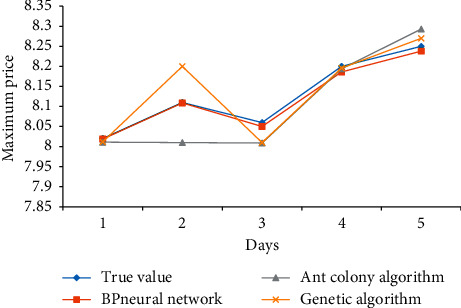
Comparison of the highest price forecast results.

The prediction results of BP neural network, ant colony algorithm, and genetic algorithm for several different indexes of Yatai Group are as follows: Figures 5–7.

The forecast results of Yatai Group's 5-day KDJ index opening price are compared in Figure 5.

From Figure 5, we can see that the BP neural network is relative to the other two algorithms, In addition to the fourth day, the prediction error is larger than that of ant colony algorithm and genetic algorithm, and its prediction value is closer to the real opening value at other times, followed by ant colony algorithm and genetic algorithm. Their errors are not much different from the real value, but they are slightly worse than BP neural algorithm.

The forecast results of Yatai Group's 5-day KDJ index closing price are compared in Figure 6.

It can be seen from Figure 6 that the error value between the daily closing value and the real value predicted by BP is the smallest, which is very close to the change curve of the real value, followed by ant colony algorithm with smaller error value, and finally genetic algorithm.

The prediction results of the highest price of Yatai Group's 5-day KDJ index are compared in Figure 7.

It can be seen from Figure 7 that the predicted value of the BP neural network is almost completely consistent with the real value the next day, but both ant colony algorithm and genetic algorithm have large error values, and their two algorithms have large error fluctuations, while the BP neural network has relatively small and stable error.

**Figure 8:**
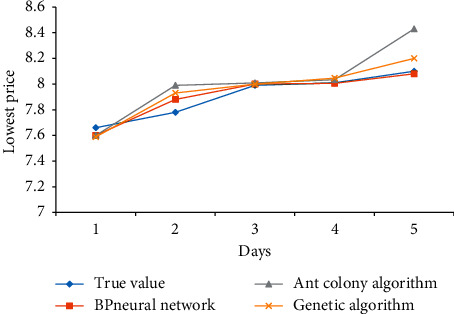
Comparison of lowest price prediction results.

The prediction results of the lowest price of Yatai Group's 5-day KDJ index are compared in Figure 8.

Through Figure 8, we can also see that the BP neural network has the smallest error value among the three algorithms, genetic algorithm at this time, and ant colony algorithm at last.

## 5. Conclusion

It is very feasible to apply the BP neural network to the research and prediction analysis of financial stability. Its three-layer structure can be arbitrarily approximated to any nonlinear continuous function, which has certain advantages in dealing with complex problems and can predict data results more accurately and reliably; he also has a high degree of self-learning and self-adaptability, which will provide stronger explanatory power than other algorithms; he also has efficient data analysis ability and can obtain strong practicability; and its fault tolerance makes it have stronger practical ability. All these advantages provide a good support for it to successfully predict financial stability with smaller errors.

## Figures and Tables

**Table 1:** Forecast data of opening price of Yatai Group's 5-day KDJ index.

Yatai	Opening price				
True value	7.66	8.01	8.04	8.10	8.21
BPneural network	7.599	7.956	8.035	8.156	8.198
Ant colony algorithm	7.588	7.920	8.030	8.144	8.193
Genetic algorithm	7.578	7.900	8.034	8.166	8.200

**Table 2:** Forecast data of closing price of KDJ index in 5 days of Yatai Group.

Yatai	Closing price				
True value	7.66	8.03	8.01	8.12	8.23
BPneural network	7.600	8.001	8.02	8.116	8.208
Ant colony algorithm	7.599	7.999	8.009	8.134	8.193
Genetic algorithm	7.588	7.930	7.999	8.146	8.200

**Table 3:** Forecast data of the highest price of KDJ index in 5 days of Yatai Group.

Yatai	Maximum price				
True value	8.02	8.11	8.06	8.20	8.25
BPneural network	8.019	8.109	8.050	8.186	8.238
Ant colony algorithm	8.011	8.010	8.009	8.194	8.293
Genetic algorithm	8.012	8.200	8.01	8.196	8.270

**Table 4:** Forecast data of the lowest price of Yatai Group's 5-day KDJ index.

Yatai	Lowest price				
True value	7.66	7.78	7.99	8.01	8.10
BPneural network	7.600	7.880	8.000	8.006	8.080
Ant colony algorithm	7.599	7.990	8.009	8.034	8.430
Genetic algorithm	7.588	7.930	7.999	8.046	8.200

## Data Availability

The experimental data used to support the findings of this study are available from the corresponding author upon request.
